# The HSP90 inhibitor Onalespib exerts synergistic anti-cancer effects when combined with radiotherapy: an in vitro and in vivo approach

**DOI:** 10.1038/s41598-020-62293-4

**Published:** 2020-04-03

**Authors:** Diana Spiegelberg, Andris Abramenkovs, Anja Charlotte Lundgren Mortensen, Sara Lundsten, Marika Nestor, Bo Stenerlöw

**Affiliations:** 1 Department of Immunology, Genetics and Pathology, Uppsala University, Uppsala, Sweden; 2 Department of Surgical Sciences, Uppsala University, Uppsala, Sweden

**Keywords:** Preclinical research, Cancer, Radiotherapy

## Abstract

Oncogenic client-proteins of the chaperone Heat shock protein 90 (HSP90) insure unlimited tumor growth and are involved in resistance to chemo- and radiotherapy. The HSP90 inhibitor Onalespib initiates the degradation of oncoproteins, and might also act as a radiosensitizer. The aim of this study was therefore to evaluate the efficacy of Onalespib in combination with external beam radiotherapy in an *in vitro* and *in vivo* approach. Onalespib downregulated client proteins, lead to increased apoptosis and caused DNA-double-strands. Monotherapy and combination with radiotherapy reduced colony formation, proliferation and migration assessed in radiosensitive HCT116 and radioresistant A431 cells. *In vivo*, a minimal treatment regimen for 3 consecutive days of Onalespib (3 × 10 mg/kg) doubled survival, whereas Onalespib with radiotherapy (3 × 2 Gy) caused a substantial delay in tumor growth and prolonged the survival by a factor of 3 compared to the HCT116 xenografted control group. Our results demonstrate that Onalespib exerts synergistic anti-cancer effects when combined with radiotherapy, most prominent in the radiosensitive cell models. We speculate that the depletion and downregulation of client proteins involved in signalling, migration and DNA repair mechanisms is the cause. Thus, individually, or in combination with radiotherapy Onalespib inhibits tumor growth and has the potential to improve radiotherapy outcomes, prolonging the overall survival of cancer patients.

## Introduction

Prognosis and overall survival of patients diagnosed with cancer has dramatically improved in the last decades. However, high initial cancer treatment response rates are often followed by the development of resistance to the therapy and eventual recurrence of the disease. Oftentimes, the recurring tumor is even more aggressive than the initial disease. Following tumor relapse, current clinical practice is sequential therapy using first and second lines of treatment. The idea is to restore treatment efficacy by switching treatment modality to counter the developing resistance. However, there is a growing body of evidence of cross-resistance between treatments, in which resistance to the second-line treatment co-develops during the first-line treatment. One strategy to avoid development of cross-resistance is to use direct combination treatment, since subpopulations of cells resistant to either single therapy would be eliminated before doubly resistant mutants emerge, increasing the likelihood of treatment success^1^. Combining anti-cancer treatments not sequentially, but simultaneously is an appealing approach to maintain clinical efficacy and to overcome the development of therapy resistance^2^. The risk of development of resistance and relapse of the disease may be further decreased by using multi-target combination approaches since they present an improved chance of affecting the complex balance and homeostasis of the entire intra- and inter cellular network compared to therapies or combinations of therapies that act on a single target.

Generally, combination treatment strategies with additive and synergistic interactions have the potential to maximize the response rate in clinical practice. Moreover, by combining additive and synergistic treatment modalities with non-overlapping toxicities, the therapeutic dosages required of each individual treatment may be lowered without compromising therapy efficacy and therefore maybe even reduce unwanted side effects. In addition, drug combinations can overcome patient to patient variability and lead to better outcomes, even if there is no drug synergy or additivity^3^.

Overexpression and or deregulation of heat shock protein (HSP) chaperones have been associated with a number of clinical conditions, including diabetes and cancer^4^. The heat shock protein 90 (HSP90) belongs to a conserved and very ubiquitous group of stress proteins that amounts to ~2% of the total cellular protein content and can be found in all cells in all organisms, from bacteria to humans. The molecular chaperone HSP90 plays a crucial role in protein-protein interactions, correct folding and transport of its client proteins as well as cell-cycle control, and protection of cells against stress and apoptotic signaling as an integral component of a chaperone complex. Cells exposed to stressors (including heat, ionizing radiation, heavy metals, acidosis or hypoxia) overexpress HSP90; and cancer cells are often addicted to increased HSP90, an adaptive response that enhances cell survival^5^.

Therefore, inhibition of HSP90 as a strategy to control cancer is appealing since it leads to downregulation of multiple oncogenic proteins and pathways simultaneously. So far 18 HSP90 inhibitors have entered clinical development^6^. However clinical outcomes have been moderately successful, mainly due to difficult formulation and adverse effects like hepatotoxicity. In 2017 alone, 17 clinical trials assessing anti-HSP90 drugs have been active, of which 7 were evaluating the inhibitor Onalespib (AT13387; NCT01712217, NCT02503709, NCT02097225, NCT02572453, NCT02381535, NCT02535338, NCT02474173)^6^.

Radiotherapy is one of the most common treatment options for cancer patient management, and approximately two thirds of all cancer patients receive radiotherapy. Despite all improvements of radiotherapy, including new modalities and optimized treatment schedules, not all patients are cured by radiotherapy and side effects can be severe and lead to lower quality of life or even secondary cancers. Several HSP90 client proteins are involved in DNA damage response (DDR) pathways, for example BRCA1, BRCA2, CHK1, DNA-PKcs, ATM, FANCA, and the MRE11/RAD50/NBN complex and inhibition of these pathways sensitize cells to radiation exposure^7,8^. Previous *in vitro* studies by our group have shown promising radiosensitizing effects of Onalespib in combination with external beam radiotherapy^9^, however radiosensitising effects of the drug have not been evaluated *in vivo*.

Consequently, in this paper we investigate combination treatment of HSP90 inhibitor Onalespib with external beam radiotherapy *in vitro* and *in vivo*, in order to further elucidate the potential mechanisms behind the proposed effects, and to assess treatment efficacy and potential toxicity of such a combination *in vivo*, using two human xenograft mouse models with different radiosensitivities. To our knowledge this is the first study evaluating potential radiosensitizing effects of Onalespib with external beam radiation in an *in vivo* setting.

## Results

### Influence on tumor cell growth and migration

**Figure 1:**
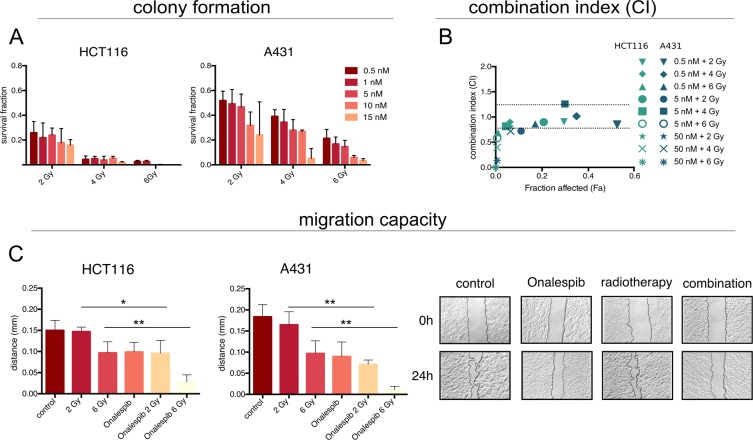
Effects on cell growth and migration. (**A**) Survival fraction measured by clonogenic assays. Radiosensitive HCT116 and resistant A431 cells were grown in monolayer and exposed to 0.5, 1, 5, 10 and 15 nM Onalespib for 10–14 days. 24 h after drug expose cells were irradiated with 2, 4 or 6 Gy. (**B**) Additive (0.8 < CI < 1.2) and synergistic (CI < 0.8) effects displayed as combination index (CI) plot for Onalespib and radiotherapy treated clonogenic assays of HCT116 and A431 cells. (**C**) Migration capacity of A431 and HCT116 cells. Cells were exposed to 2 doses of radiotherapy (2 and 6 Gy) and to 500 nM Onalespib n > 3, error bars: SD. Left) migration distance, Right) Representative pictures of scratch area at 0 and 24 h, HCT116 cells treated with 500 nM Onalespib and a radiation dose of 2 Gy. Both A431 and HCT116 cells treated with 6 Gy and Onalespib lost the migrating potential.

To investigate the treatment efficacy of Onalespib and/or radiotherapy, colony formation assays were performed, as displayed in Fig. 1A. The survival fractions of HCT116 and A431 cells were reduced with increasing doses of long time exposure (10–14 days) to Onalespib and radiation doses of 2, 4 or 6 Gy. HCT116 cells were more sensitive to both the mono- and combination treatments in comparison to A431 cells. All tested combinations displayed additive or synergistic effects in HCT116 cells with combination indexes (CI) below 0.9 (Fig. 1B). Treatment with 0.5 nM and radiotherapy of 2, 4 or 6 Gy as well as 5 nM Onalespib and 2 and 4 Gy resulted in mainly additive effects for A431 cells, whereas concentrations of 5 nM and 6 Gy and higher showed synergistic interactions (Fig. 1B).

The capacity of cancer cells to migrate after drug and radiotherapy was investigated with wound healing assays. Vehicle treated (control) HCT116 cells migrated a distance of 0.18 mm within 24 h, while A431 migrated a distance of 0.15 mm. Onalespib treatment of HCT116 and A431 cells reduced the migration rate by 51% ± 11% and 34% ± 9%, respectively. Radiotherapy with 2 Gy did not result in a significant effect on HCT116 and A431 cells. A radiation dose of 6 Gy reduced the migration rate of HCT116 and A431 cells by 47% ± 10% and 35% ± 12%, respectively. The largest inhibitory effect was observed in the combination treated samples. In the combination group the migrating capacity was reduced by 94% ± 9% for HCT116 by 81% ± 10% for A431 cells (Fig. 1C).

### Protein expression and apoptosis analysis

**Figure 2:**
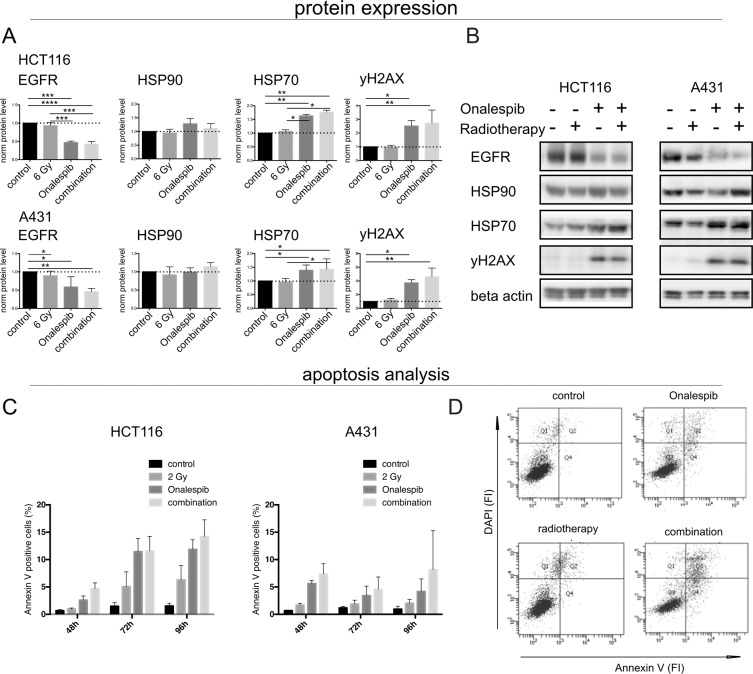
(**A**,**B**) Protein expression and apoptosis analysis. Western blot analysis of HCT116 and A431 cells. (**A**) Expression analysis of the above-mentioned proteins for HCT116 and A431 (normalized density), n > 3 error bars = SD. **(B)** Representative bands of the expression levels of EGFR, HSP90, HSP70, γH2AX and the housekeeping protein (loading control) beta actin 24 h after treatment. Images display cropped blots gathered with exposure times applied equally across the entire image, however different for different membrane fragments of the assessed proteins. All images are taken from membranes of the same lysates. (C,D) Flow cytometry analysis, of Annexin V expression. (**C**) Annexin V expression plotted as % positive cells after 48, 72 and 96 h treatment. n > 3, error bars = SD. (**D**) Representative image of Annexin V and DAPI analysis as fluorescence intensity (FI) for A431 cells at 48 h.

Western blotting was used to study the expression of the molecular chaperones HSP90 and HSP70 as well as the HSP90 client protein EGFR and the DNA double strand marker γH2AX post drug and radiation exposure (Fig. 2A,B). The expression levels of HSP90 were not significantly changed after drug or radiation exposure. The expression level of the co-chaperone HSP70 were increased for both the Onalespib and combination treated group by 63 ± 3% to 76 ± 4% for HCT116 and for about 47 ± 9% to 70 ± 20% for A431 cells. The expression of the cell surface growth factor receptor EGFR was significantly downregulated in both cell lines in the Onalespib and the Onalespib combined with radiation treatment groups. In HCT116 samples the receptor expression was reduced by 52 ± 2% and 57 ± 3%, respectively. Radiation treatment alone had no significant effect on EGFR expression in HCT116 cells (reduction of 7 ± 4%), whereas a minor reduction was seen in A431 cells (17 ± 4%). The DNA damage marker γH2AX was significantly increased in all Onalespib-treated groups for both cell lines. Onalespib-treated HCT116 cells demonstrated a 2.5 times higher expression of the marker (152 ± 20%), whereas A431 cells demonstrated an increase of 232 ± 19%. The expression in the combination treated cells was increased by 198 ± 58% for HCT116 and 235% ± 36% for A431 cells.

To investigate the effect of Onalespib combined with radiotherapy on survival mechanisms such as apoptosis, flow cytometry with Annexin V analysis was performed at 48 h, 72 h and 96 h after drug exposure (Fig. 2C,D). HCT116 cells demonstrated a significant increase in Annexin V levels after 72 h and 96 h following the treatment with Onalespib. This effect was further potentiated by the combination treatment of Onalespib and radiation treatment with 2 Gy. Staining for Annexin V peaked at 96 h for both HCT116 and A431 cells in the drug and combination group. However, Annexin V staining was more pronounced in radiosensitive HCT116 cells with 14% of population being positive while population of A431 cells exhibited 8% positivity.

### DNA repair foci

**Figure 3:**
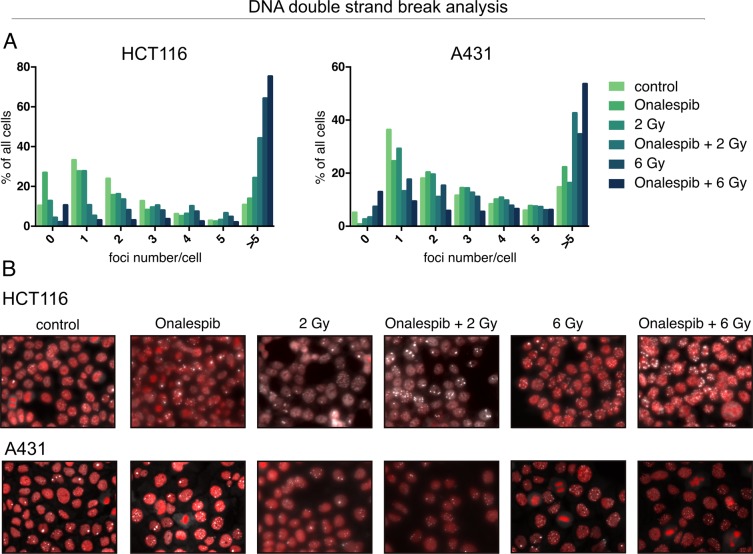
Onalespib increases the retention of radiation-induced 53BP1 foci. Confocal microscopy of DNA repair foci, stained for 53BP1. (**A**) 53BP1 foci scoring in HCT116 and A431 cells 24 h after exposure to 500 nM Onalespib and radiotherapy with 2 and 6 Gy. The graph shows a percentage (%) of cells containing 1, 2, 3, 4, 5, and >5 foci/cell. For each treatment group more than 50 cells were scored. (**B**) Representative images of HCT116 and A431 cells 24 h after the different treatments.

DNA repair capacity after treatment with Onalespib and radiotherapy was studied by analysis of the DSB marker 53BP1on the tumor cell lines HCT116 and A431 (Fig. 3A,B). The cells were pre-treated with radiation and drug 24 h prior analysis. Generally, untreated HCT116 and A431 cells demonstrated on average 1–2 foci/cell and 1 foci/cell respectively. HCT116 and A431 treated with Onalespib alone demonstrated an increase in foci number, about 30% more HCT116 cells contained >5 foci/cell compared to the control cells, and 50% more for A431. Radiotherapy increased the foci number in a dose dependent manner: After a radiation dose of 2 Gy 24.3% of HCT116 and 16.3% of A431 cells had >5 foci/cell, and after 6 Gy 64.3% of HCT116 and 34.7% of A431 cells displayed >5 foci/cell. The highest fraction of cells containing persisted damage with >5 foci was observed in the combination treatment group with the highest radiation dose of 6 Gy. Here, 75.3% of all HCT116 and 53.6% of A431 cells contained >5 foci/cell.

### Mouse xenografts

**Figure 4:**
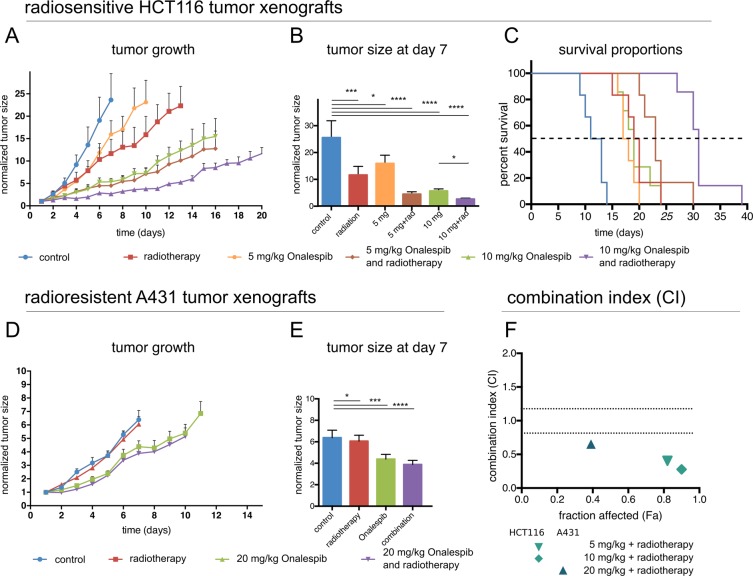
*In vivo* mouse xenograft study (**A**–**C**) with radiosensitive HCT116 and (**D**,**E**) radioresistant A431 tumors. (**A**) HCT116 tumor growth over time (n > 6 per group, error bars = SD). HCT116 xenografts followed over time, until the first animal in each group was sacrificed. (**B**) HCT116 tumor size on day 7 (one week after treatment start). (**C**) Survival proportions of the different treatment groups. Statistical significance *P = 0.05, n > 6, error bars = SD. (**D**) A431 tumor growth over time (n > 4 per group, error bars = SD). A431 xenografts followed over time, until the first animal in each group was sacrificed. (**E**) A431 tumor size on day 7 (one week after treatment start). (**F**) Synergistic effects displayed as combination index (CI) plot for the combination of Onalespib and radiotherapy treated HCT116 and A431 tumor xenografts (CI < 0.8).

The potential radiosensitizing effect of Onalespib was tested in small animal models xenografted with either radiosensitive HCT116 or radioresistant A431 cells (Fig. 4A–F). Mice in the radiotherapy and drug- and radiotherapy group received a total dose of 6 Gy. Administration of drug and/or radiotherapy did not have an effect on the animal weight (data not shown). The fastest growing malignancies were observed in the control group for both HCT116 and A431 xenografts. One week after treatment start HCT116 control tumors had been growing to a 20 times bigger tumor volume than HCT116 tumors in the 3 × 10 mg/kg Onalespib and radiotherapy combination group (Fig. 4A,B). The tumor volume of monotherapy groups was significantly smaller than the controls, however not as pronounced as in the combination group with the highest Onalespib dose. After one week, radiation treated tumors grew to approximately half of the size of control tumors, and 3 × 10 mg/kg Onalespib treated tumors to one fourth of the controls. Furthermore, HCT116 control mice had a median survival of 9.5 days. The maximum survival was 14 days in this group (Fig. 4C). The radiotherapy group and the 3 × 5 mg/kg Onalespib group presented a median survival of about 17.7 days, and the 10 mg/kg group 19 days. The maximum survival was 20 and 24 day, respectively. Animals in the combination group demonstrated a clear survival advantage, with a median survival dependent on the Onalespib dose. Mice receiving a combination treatment with 3 × 5 mg/kg had an increase in median survival by about 2.4 times (23 days) and with 3 × 10 mg/kg by a factor of 3.3 (31 days) compared to control animals. Maximum survival was 30 and 39 days, respectively.

Mice carrying human A431 xenografts responded differently to the radiotherapy compared to HCT116 tumors (Fig. 4D,E). A radiotherapy with 3 times 2 Gy had no beneficial effect on A431 xenografts, the tumor size was approximately the same as in the control animals. Onalespib treatment reduced the tumor size significantly by 32% and the combination of Onalespib and radiotherapy by 41% at 7 days after treatment start compared to the control group. Similar to HCT116 xenografts, mice carrying A431 tumors did not show any changes in body mass of and did not show any other adverse effects (data not shown). All tested combinations displayed synergistic effects with combination indexes (CI) below 0.8 (Fig. 4F). The survival data of HCT116 and A431 xenografts is summarized in Table 1.

**Table 1:** Median survival and maximum survival (days) of HCT116 and A431 xenografted animals.

HCT116	median survival (days)	maximum survival (days)
control	9.5	14
radiotherapy	17	24
5 mg/kg Onalespib	17.7	20
5 mg/kg Onalespib and radiotherapy	23	30
10 mg/kg Onalespib	19	24
10 mg/kg Onalespib and radiotherapy	31	39
**A431**	**median survival (days)**	**maximum survival (days)**
control	8.5	13
radiotherapy	9	11
20 mg/kg Onalespib	12	13
20 mg/kg Onalespib and radiotherapy	12	13

### *Ex vivo* IHC

**Figure 5:**
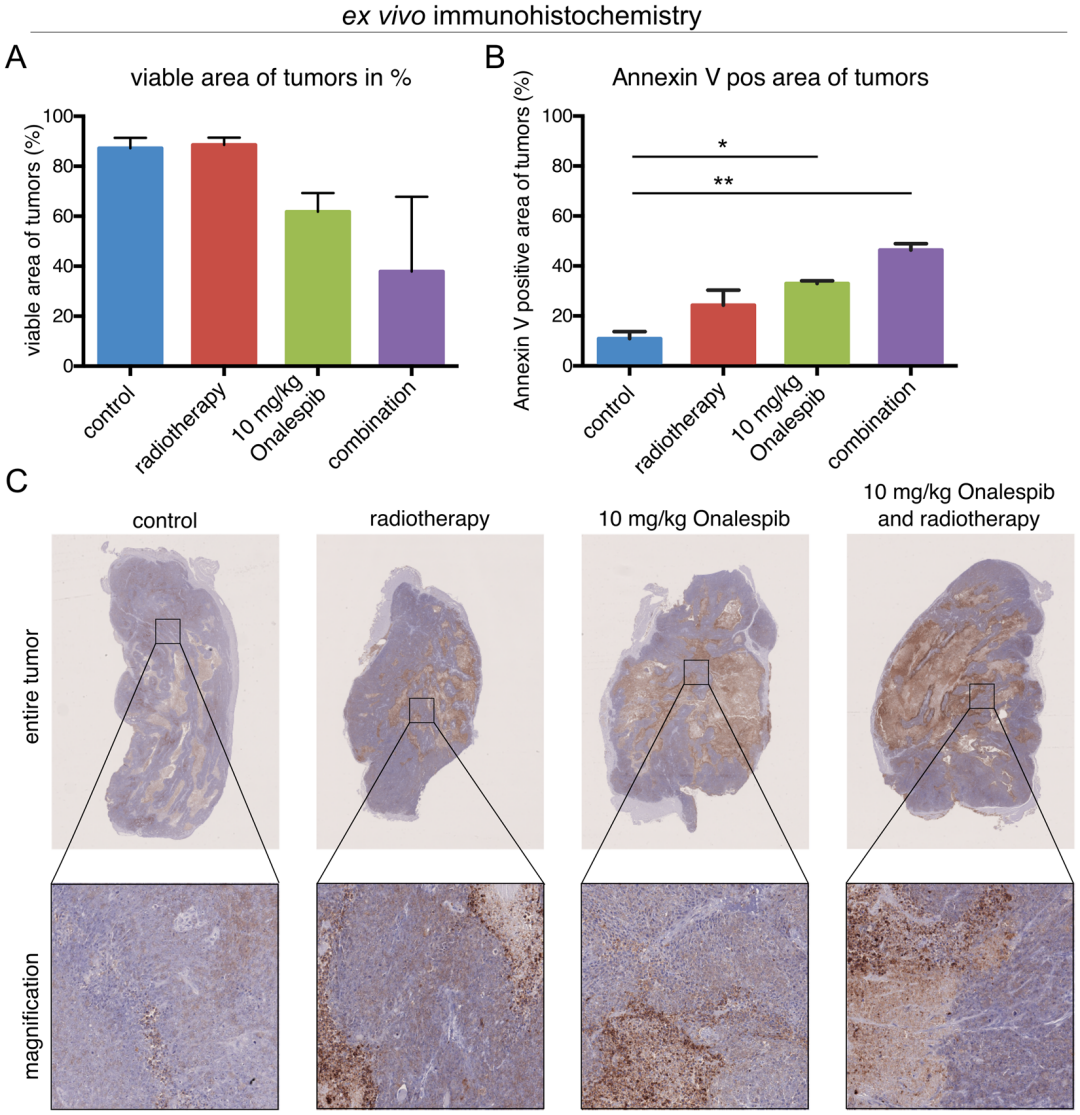
*Ex vivo* immunohistochemistry analysis of HCT116 tumors. (**A**) Score of viable area (non-necrosis) within the tumor after treatment with Onalespib, radiotherapy and or both. (**B**) Scoring of Annexin V expression in the tumors after treatment. (**C**) Representative images of HCT116 tumors stained with Annexin V and hematoxylin. The upper row shows the entire tumor, while the lower row is a magnification of the marked area above. n > 2, error bars = SD.

To study the effect of the single treatments and the combination of radiotherapy and Onalespib on the xenografted tumors and healthy organs, *ex vivo* immunohistochemistry was performed. HCT116 tumors of the different treatment groups were analyzed when reaching a size of 1000 mm^3^. Even though the tumor size was comparable, the portion of viable tissue vs. necrotic tissue differed between groups. More than 80% of the tumor tissue receiving placebo or radiotherapy was viable, whereas HCT116 tumors treated with 10 mg/kg Onalespib and 10 mg/kg Onalespib and radiotherapy showed a reduction in viable area of 29% ± 7% and 57% ± 24%, respectively (Fig. 5A,C). Furthermore, combination treated and Onalespib treated tumors displayed a significant increase in the apoptosis marker Annexin V (Fig. 5B,C). About 10% of the control tumors scored positive for Annexin V. This number was increased by a factor of 3.3 (±0.5) for Onalespib (33%) and by a factor of 4.6 (±0.9) for the combination group (46%). Manual scoring of the tumor sections revealed that the Annexin V stained areas correlated with viable tumor areas, with highest expression in the radiation and combination group (Supplementary Table 1). Further, Ki67 expression did not differ significantly between the treatment groups (Supplementary Table 2).

## Discussion

In this study we investigated the efficacy of the new anti-HSP90 drug Onalespib in combination with external beam radiation therapy. Previous *in vitro* assays by our group showed promising radiosensitizing effects of Onalespib, however the combination of Onalespib and external radiotherapy has to our knowledge never been studied before in an *in vivo* setting. Consequently, this is the first study investigating these effects in *in vivo*, confirming that Onalespib together with external radiotherapy kills cancer cells more effectively than single treatments.

As evident from the survival and viability analyses, HCT116 cells were significantly more sensitive to the single and combination treatments in comparison to A431 cells (Fig. 1A,B), which is in line with previous studies, where radiotherapy and Onalespib treatment acted in an additive manner for A431 cells and in a synergistic for HCT116^9^. Onalespib demonstrated an effect on both cell lines, however this effect was more pronounced in HCT116 cells. One reasonable explanation for the lesser efficacy in A431 could be the increased radioresistance of A431 cells compared to HCT116, which has been described earlier^9^. Furthermore, synergistic radiosensitizing effects of Onalespib were recently observed in a study of neuroendocrine tumor cells in which Onalespib was combined with peptide receptor radionuclide (PRRT) therapy in multicellular spheroid models^10^.

Cell migration analysis revealed that radiotherapy alone at doses as low as 2 Gy reduced the migration capacity by 23% in HCT116, whereas it was not affected for A431 cells. This is probably due to the higher radiation tolerance of A431 cells. However, Onalespib treatment and radiotherapy with 6 Gy blocked the ability to migrate in both cell lines to high extent. Both intracellular and extracellular HSP90 play a significant role in regulating cell migration and angiogenesis^11,12^. Today it is known that cell migration is not only an integral part initiating metastatic growth but also is required at every other step of the metastatic cascade^13^. Further, given that the majority of cancer-related deaths are due to metastatic disease, blocking the migration capacity should be a major focus of cancer research and demonstrates the superior efficacy of the combination therapy compared to the monotherapies.

Protein expression levels post drug and radiation exposure were assessed by Western blotting (Fig. 2A,B). The expression of the cell surface growth factor receptor EGFR was significantly downregulated in modest EGFR-expressing HCT116 (EGFR++) and EGFR-overexpressing A431 (EGFR+++) cells in the Onalespib and the Onalespib combined with radiotherapy group. The receptor expression was reduced by more than 50% in the named groups. The downregulation of HSP90 as well as its client proteins is a very attractive measure for treatment efficacy *in vivo* and expression levels of EGFR or HER2 among others have been assessed for treatment response monitoring with molecular imaging techniques like PET and SPECT cameras^14–16^.

To function properly, HSP90 requires the collaboration with a subset of co-chaperones assembling a chaperone complex also called HSP90 chaperone machinery, including HSP70^17^. HSP90 and HSP70 interact indirectly through the co-chaperone HOP. As stated previously^18,19^, we observed an increase of the co-chaperone HSP70 after HSP90 inhibition. HSP70 upregulation proves the inhibition of HSP90 by Onalespib, albeit to a larger extent in HCT116 cells. The DNA damage marker γH2AX was significantly increased in all drug-treated groups in both cell lines. Our findings demonstrate that Onalespib treatment increases the amount of DNA double strand breaks, by either a direct effect on the DNA and repair or indirectly by downregulation of signaling and DNA repair proteins, which has been reported earlier^7^. The fact that no increase in DSBs was seen in the radiotherapy group is due to the assessed time point. Usually, the expression of γH2AX peaks around 1 h after radiation treatment, so at 24 h post radiotherapy most cancer cells have repaired the inflicted damage. Microscopic analysis of the DSB marker 53BP1 was in line with the analysis of γH2AX using Western blot, demonstrating reduced DNA repair in the treated samples or that additional DSBs are generated in the combination group. When scoring the amount of DSB foci after the treatments, cells containing more than 5 DSBs are predominant in the combination group. Even untreated cells show some DSBs, which is commonly seen in cells experiencing genomic instability like cancer cells. These findings indicate that untreated cells had a sufficient DDR and that combination treated cells had an inferior DNA repair compared to control or monotherapies. Although Onalespib has been previously shown to downregulate the key DNA repair proteins ATM and DNA-PKcs^9^, the present data do not suggest a strong effect on DSB repair. However, downregulation of DNA-PKcs can lead to sensitization of tumor cells to radiation, via accumulation of cells in mitosis, independent on its role in DSB repair^20^.

Flow cytometry analysis demonstrated a significant increase in Annexin V after 72 h and 96 h post Onalespib treatment. HSP90 is able to block apoptosis by interfering with caspase activation upon a variety of cellular stresses, including reactive oxygen species (ROS) or DNA damage. In turn, depletion of HSP90 can increase the cells’ sensitivity to apoptotic inducements^21^. Our results are in line with these findings and demonstrate that apoptotic stimuli triggering radiotherapy (like ROS) in combination with HSP90 inhibition lead to significant higher apoptosis response as compared to apoptosis levels of the control and monotherapy groups.

To summarize, our *in vitro* assessments demonstrated that HSP90 inhibition potentiates radiotherapy in HCT116 cells, by mechanisms such as increased DNA damage, decreased metabolic rate, increased apoptosis and reduced migration capacity. The effects were also observed for A431 cells, although the radioresistant nature of these cells required higher radiation doses in some cases in order to obtain an effect.

To further evaluate the efficacy of Onalespib and radiotherapy in a more clinically relevant setting the combination was tested in mice xenografted with HCT116 and A431 tumors. Animals were treated with repeated doses of Onalespib and fractionated radiotherapy of 3 × 2 Gy, as 2 Gy is a common dose for fractionated radiotherapy in clinical practice. Shorter fractioning times were used for HCT116 tumors than A431 to compensate for the more rapid doubling time for this cell line. In HCT116 cells monotherapy of Onalespib with 5 mg/kg significantly reduced tumor growth, prolonged median and overall survival. Monotherapy with Onalespib of 10 mg/kg was able to more than double median survival. The combination group survived the longest, 3.3 times longer than compared to untreated controls, verifying the *in vitro* results. In our *in vivo* investigation no acute adverse effects, e.g., weight loss, were observed at any point in the study for neither HCT116 nor A431 xenografted animals, demonstrating a good tolerability of the treatment.

Tumors treated with Onalespib and with Onalespib and radiotherapy had a drastic reduction of viable cells and significantly more apoptosis compared to untreated control tumors. These results are impressive, since the tumors were taken when they reached a size of 1000 mm^3^, which was on all cases at least more than one week after end of the treatment, further validating the superior effects of the combination treatment.

Mice carrying A431 xenografts benefited from the Onalespib treatment as well. Repeated doses of 20 mg/kg reduced the tumor growth by 32% compared to controls. The same radioresistance of A431 as seen in the *in vitro* assays was observed, with no significant effect on tumor growth. Here, a higher radiation dose, increased numbers of fractions or shorter time between fractions (as for HCT116) might have given a better outcome. Consequently, there was no benefit of combination treatment at these settings. For this investigation we chose rather conservative doses of Onalespib and radiotherapy. Recent studies with Onalespib in xenografted mice used much higher doses (30–70 mg/kg, once or twice a week) and were able to show an elongated overall survival and a good tolerance of the drug^22–24^.

Several HSP90 inhibitors are currently studied and have demonstrated antitumor effects and chemo- or radiation sensitizing effects. Onalespib for example, has been found to sensitize malignant glioma cells to temozolomide. The HSP90 antagonist ganetespib, has recently been investigated for sensitizing effects in ovarian cancer and pancreatic cancer cells to chemotherapy^25^ and radiochemotherapy^26^, respectively. The HSP90 antagonist NVP-AUY922 potentiated anti-cancer effects of a BCL-2 inhibitor in small cell lung cancer^27^ and demonstrated synergy in combination with trastuzumab in breast cancer^28^. We believe that it is not a coincidence that so many studies currently investigate combination therapies with HSP90 inhibition. It shows that this strategy is a novel and very attractive concept, valid for several malignant diseases, and might change in future the current therapeutic approaches.

To conclude, we have for the first time demonstrated the great potential of utilizing HSP90 inhibition with Onalespib and external radiotherapy *in vivo*, and have identified increased apoptosis, loss of migration capacity as well as insufficient DDR as the most likely mechanisms behind the effects. These are very promising results which sets the stage for further clinical investigations, and eventually potential introduction in clinical use.

## Materials and Methods

### Cell lines

The radiosensitive human adenocarcinoma cell line HCT116, RRID:CVCL_0291, (radiosensitivity previously assessed by clonogenic survival assays: Survival fraction (SF)_2Gy_ 0.26, SF_4Gy_ 0.06^20^, doubling time of about 17 h^29,30^) and the radioresistant human squamous cell carcinoma cell line A431, RRID:CVCL_0037, (radiosensitivity previously assessed by clonogenic survival assays: SF_2Gy_ 0.55, SF_4Gy_ 0.25^20^, doubling time 21–30 h^31,32^) were obtained from American Type Culture Collection, ATCC (Manassas, VA, USA). HCT116 were cultured in McCoy's cell culture medium (Biochrom Kg, Berlin, Germany), supplemented with 9% fetal calf serum (Sigma Aldrich), 2 mM L-glutamine, and antibiotics (100 IU penicillin and 100 μg/mL streptomycin) (Biochrom Kg). A431 cells were cultured in Ham's F10 medium (Biochrom Kg), with the same supplements as described above. Cells were incubated at 37 °C in an atmosphere containing 5% CO_2_. All experiments were performed with mycoplasma-free cells and all cell lines were cultured for <6 months after purchase.

### Drug formulation and radiation treatment

Drug formulation for *in vitro* studies: 5 mg Onalespib (AT13387, Selleckchem) was dissolved in 200 µL DMSO and further diluted in cell culture medium before use. For *in vivo* studies: 5 mg Onalespib was dissolved in 200 µL DMSO and further diluted in 17. 5% β-Cyclodextrin (Sigma-Aldrich).

Irradiation: *In vitro* and *in vivo* experiments were irradiated with 137-Cs γ-rays using a Gammacell 40 Exactor (Best Theratronics Gammacell 40 Exactor, dose rate 1 Gy/min). For *in vivo* studies: Local irradiation of tumor xenografts (posterior leg) was achieved with specific collimators (Best Theratronics) resulting in a 3-cm radiation field.

### Colony formation assays

Colony formation assays were prepared as described in^33^. In short, HCT116 and A431 cells were pre-plated and after 24 h exposed to Onalespib concentrations of 0.5, 1, 5, 10 and 15 nM. Radiotherapy of 2, 4 or 6 Gy was performed 1 h after drug expose. After incubation of 10–14 days at 37 °C, cells were rinsed in PBS and fixated in 90–95% ethanol, followed by crystal violet stain. Colonies of >50 cells were scored manually and Plating efficiency (PE) and SF were calculated as described in^33^.

### Migration assays

The cell migration ability of HCT116 and A431 cells was studied using a scratch assay. Here, cancer cells grown in 6-well plates and were washed and incubated with complete medium, containing 500 nM Onalespib. 1 h after drug incubation, cells were irradiated with a radiation dose of 2 or 6 Gy. Using a p10 pipette tip, a narrow area of the cancer cells confluent was scratched off. Images from the same scratch area were taken at the timepoints 0 h and 24 h. The migration distance in mm was calculated as (width of the scratch at time 0 – width of the scratch at 24 h)/2.

### Western blotting

Whole cells lysates of HCT116 and A431 were prepared according to standard procedures and the protein concentration measured with an IMPLEN NanoPhotometer P-class P360. The lysates were run on a 3–8% Bis-Tris SDS-PAGE (Life Technologies) and transferred by wet blotting (overnight, 4 °C) to a PVDF membrane (Merck/Millipore). After 1 h of blocking (PBS, 5% BSA) the membrane was cut horizontally in smaller fragments and incubated with primary antibodies over night at 4 °C. Primary antibodies used: rabbit anti-HSP90 antibody (ab13495, abcam, 1/30000), mouse anti-phospho-histone H2A.X (Ser139) antibody clone JBW301-I (Merck/Millipore, 1 μg/mL), mouse monoclonal anti-beta-actin antibody (A5316, Sigma Aldrich, 1/10000), rabbit anti-EGFR antibody [EP38Y] (ab52894, abcam, 1/5000). Next day, the membrane was rinsed in 3 times for 5 min in PBS with 1% Tween-20 and incubated with respective and anti-mouse or anti rabbit secondary antibodies with HRP-label (Life technologies). Membrane fragments were incubated with SuperSignal West Pico PLUS chemiluminescent substrate (Thermo Scientific) and bands were visualized with a SuperCCD HR camera (Fujifilm). ImageJ (NIH) was used for quantitative analysis were relative protein levels were compared with the loading control levels from the same lysate with the same treatment.

### Flow cytometry

HCT116 and A431 cells were incubated with 500 nM Onalespib or complete media (control) for 24 h, followed by irradiation with 2 Gy. Live cell analysis via flow cytometry was performed 48 h, 72 h as well as 96 h after radiation. Here, cells were washed in PBS and incubated in Annexin V binding buffer (140 mM NaCl, 2.5 mM CaCl_2_, 1 mM MgCl_2_, 10 mM HEPES) for 5 minutes. 5 µL of Annexin V (APC conjugated; PARTEC; 05–7–490 A or Alexa Fluor 350 conjugated; ThermoScientific; A23202) were then added to each sample at least 15 minutes before analysis. 30 seconds before a sample was run, 0.5 µg/mL DAPI (Thermo Scientific; 62248) was added. Analysis was performed on a LSR II SORP flow cytometer (BD).

### Confocal microscopy

Chamber slides were seeded with HCT116 and A431 and incubated with 500 nM Onalespib or complete media (control). After 24 h, slides were irradiated with 0 (control), 2 or 6 Gy. The cells were washed in cold PBS and fixated in ice-cold 99.5% ethanol 24 h post irradiation. Next, the slides were dipped into ice-cold acetone for approximately 10 seconds, air dried and blocked (10% FBS) for 1 h in room temperature. The cells were then stained with 53BP1 (rabbit primary antibodies; dilution 1:1000, 50 µL/well; solution of 1% FBS in 1x PBS) overnight in 4 °C. The next day, the slides were washed 3 × 5 minutes in 1xPBS and incubated for 60 minutes in RT with the Alexa Fluor 488; ab150117; dilution of 1:400. After a washing step with 1x PBS (3 × 5 minutes) the cells were incubated with DAPI (1 µg/mL) for 2 minutes and washed with MQ water (2 × 5 minutes), air dried and mounted in VectraShield (Vectorlabs). The images were acquired using Axioimager or LSM 700 (Ziess, Jena, Germany). The foci then were scored manually or with custom macro in ImageJ (NIH).

### Mice xenograft study

Female nu/nu Balb/c mice were housed under standard laboratory conditions and fed ad libitum. All experiments complied with Swedish law and were performed with permission from the Uppsala Committee of Animal Research Ethics, with maximum allowed tumor size 1000 mm^3^. Licensee for Uppsala University: Vice-rector Stellan Sandler, applicant Marika Nestor, ethical permission number C9/16. Tumor xenografts were formed by subcutaneous inoculation of approximately 1 × 10^6^ HCT116 or 5 × 10^6^ A431 cells suspended in 100 μL serum free cell culture medium in the right posterior leg. After approximately 10 days tumors had established (>80 mm^3^) and the treatment schedule started (animal age of approximately 9 weeks). HCT116 xenografted mice were divided into the following treatment groups: (i) control (N = 10), (ii) radiotherapy (N = 10) 3 × 2 Gy, (iii) Onalespib (N = 10) 3 × 10 mg/kg Onalespib, (iiii) Onalespib and 3 × 10 mg/kg combined with 3 × 2 Gy (N = 10), (iv) Onalespib 3 × 5 mg/kg (N = 10), (v) Onalespib 3 × 5 mg/kg combined with 3 × 2 Gy (N = 10). Mice were injected intraperitoneally with 100 µL Onalespib or (controls and radiation groups) with 100 µL 17.5% β-Cyclodextrin (Sigma-Aldrich, Sweden) on day 1, 2, and 3. Radiotherapy was given 6 h after the drug treatment, performed under anesthesia on day 1, 2, and 3.

A431 xenografted mice were divided into the following treatment groups:

(i) control (N = 10), (ii) radiotherapy (N = 9) 3 × 2 Gy, (iii) Onalespib (N = 10) 3 × 20 mg/kg Onalespib, (iiii) Onalespib and 3 × 20 mg/kg combined with 3 × 2 Gy (N = 9).

Mice were injected intraperitoneally with 100 µL Onalespib or (controls and radiation groups) with 100 µL 17.5% β-Cyclodextrin (Sigma Aldrich, Sweden) on day 1, 3, and 5. Radiotherapy was given 6 h after the drug treatment, performed under anesthesia on day 1, 3, and 5. Tumor size was measured every day with a digital caliper and survival analysis was performed after reaching the study endpoint of 1000 mm^3^ tumor size. At the endpoint, animals were euthanized with a mixture of ketamine and xylazine followed by heart puncture.

### Immunohistochemistry

Xenografted HCT116 tumors were dissected at a size of about 1000 mm^3^ and directly fixated in 36% formaldehyde. The tumors were paraffin-embedded, sectioned and deparaffinized. Pretreatment of tumor tissue with citrate pH 6 (DAKO, item number S2031) or Tris-EDTA buffer (DAKO, S2367) was performed in a pressure-cooker at 98 °C for 40 minutes. Sections were immunostained with anti-Ki67 (DAKO, M7240) and anti-Annexin V (abcam, ab108321, rabbit) afterwards detected by MACH3 HRP-Polymer kit (Biocare Medical) and counterstained with IntelliPATH Hematoxylin (Biocare Medical). The area and intensity of the staining were scored with a custom macro in ImageJ (NIH, USA). The ImageJ Colour Deconvolution plugin (H DAB setting) was used to separate the DAB signal for hematoxylin staining. Afterwards positive DAB pixels were thresholded and related to total tumor area.

For comparison between viable area, Annexin V and Ki67, a manual, blinded scoring procedure was used. 4 areas of 0.2 mm^2^ viable areas per section were scored. For Annexin V, the percentage of positive area was estimated by visual inspection. For Ki67, the number of positive cells were manually counted for each section. Each scoring area contained approximately 2000 cells.

### Statistical analysis

The data received from the experiments have been processed in Microsoft office Excel for Mac Version 16.16.2 and all graphs have been plotted in GraphPad Prism 6 for Mac OS X. Results of the viability, migration assays and western blot analysis was evaluated by one-way ANOVA with Turkey's posttest in GraphPad Prism 6. The combinative effects of Onalespib and radiotherapy in *in vitro* clonogenic assays and *in vivo* experiments were analysed by the Chou-Talalay-method^34^ by the software CompuSyn 3 developed by Nick Martin of MIT, Cambridge, MA. The fraction affected (Fa) was calculated and operates as the percent growth inhibition and CI represents the combination index. A CI of ≤0.8, and ≥1.2 indicates synergism and antagonism, respectively. A CI > 0.8 < 1.2 indicates additive effect.

The data that support the findings of this study are available from the corresponding author upon reasonable request.

## Supplementary information

Supplementary information 1.

Supplementary information 2.
