# Supplementary material for: The HSP90 inhibitor Onalespib exerts synergistic anti-cancer effects when combined with radiotherapy: an in vitro and in vivo approach
Source: Sci Rep. 2020 Apr 3;10:5923. doi: 10.1038/s41598-020-62293-4 (PMC7125222; doi:10.1038/s41598-020-62293-4)

Supplementary information

The HSP90 inhibitor Onalespib exerts synergistic anti-cancer effects when combined with radiotherapy: an in vitro and in vivo approach

Diana Spiegelberg, Andris Abramenkovs, Anja Charlotte Lundgren Mortensen, Sara Lundsten, Marika Nestor, Bo Stenerlöw

Supplementary information

Western blot membranes

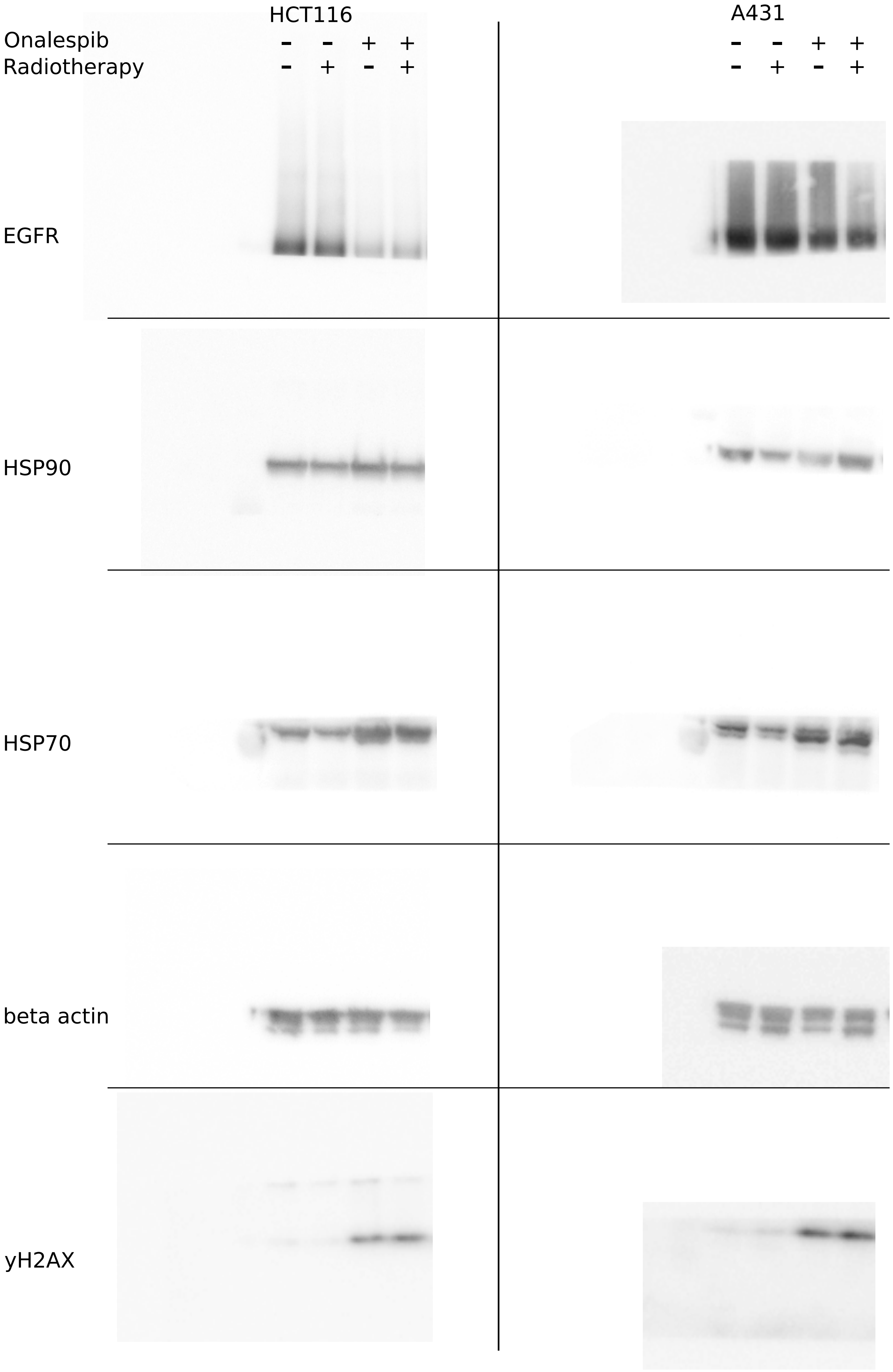

Supplement: Supplementary file 2 — Supplementary information 2. [file 41598_2020_62293_MOESM2_ESM.pdf]
